# SNP-based mixed model association of growth- and yield-related traits in popcorn

**DOI:** 10.1371/journal.pone.0218552

**Published:** 2019-06-25

**Authors:** Gabrielle Sousa Mafra, Antônio Teixeira do Amaral Júnior, Janeo Eustáquio de Almeida Filho, Marcelo Vivas, Pedro Henrique Araújo Diniz Santos, Juliana Saltires Santos, Guilherme Ferreira Pena, Valter Jario de Lima, Samuel Henrique Kamphorst, Fabio Tomaz de Oliveira, Yure Pequeno de Souza, Ismael Albino Schwantes, Talles de Oliveira Santos, Rosimeire Barbosa Bispo, Carlos Maldonado, Freddy Mora

**Affiliations:** 1 Laboratório de Melhoramento Genético Vegetal, Centro de Ciências e Tecnologias Agropecuárias (CCTA), UENF, Campos dos Goytacazes, Rio de Janeiro, Brazil; 2 Bayer, Coxilha, Rio Grande do Sul, Brazil; 3 Universidade Estadual de Mato Grosso, Alta Floresta, Mato Grosso, Brazil; 4 Universidad de Talca, Talca, Chile; INRA France, FRANCE

## Abstract

The identification of the genes responsible for complex traits is highly promising to accelerate crop breeding, but such information is still limited for popcorn. Thus, in the present study, a mixed linear model-based association analysis (MLMA) was applied for six important popcorn traits: plant and ear height, 100-grain weight, popping expansion, grain yield and expanded popcorn volume per hectare. To this end, 196 plants of the open-pollinated popcorn population UENF-14 were sampled, selfed (S_1_), and then genotyped with a panel of 10,507 single nucleotide polymorphisms (SNPs) markers distributed throughout the genome. The six traits were studied under two environments [Campos dos Goytacazes-RJ (ENV1) and Itaocara-RJ (ENV2)] in an incomplete block design. Based on the phenotypic data of the S_1_ progenies and on the genetic characteristics of the parents, the MLMA was performed. Thereafter, genes annotated in the MaizeGDB platform were screened for potential linkage disequilibrium with the SNPs associated to the six evaluated traits. Overall, seven and eight genes were identified as associated with the traits in ENV1 and ENV2, respectively, and proteins encoded by these genes were evaluated for their function. The results obtained here contribute to increase knowledge on the genetic architecture of the six evaluated traits and might be used for marker-assisted selection in breeding programs.

## Introduction

In maize (*Zea mays* L.), the economically most important traits are usually quantitatively inherited, and their genetic basis is attributed to polygenes, as well as to the interaction effects between genes and/or between genes and environment [1]. Because *Z*. *mays* has been used as a model in classical genetics and cytogenetics, it has significantly contributed to the understanding of fundamental processes such as reproduction and photosynthesis, among others [2].

Association studies have been successfully used to identify the genetic basis of quantitative traits, allowing not only the identification of quantitative trait loci (QTLs), but also the identification of candidate genes based on statistical significant differences between markers and phenotypes [3]. Single nucleotide polymorphism (SNP) markers contributed to expand the knowledge of the genetic structure and diversity of maize populations. The use of these molecular markers has become fundamental in SNP-based association studies [4–6]. In maize, association studies have allowed the identification of several important traits for agriculture since the release of the B73 maize reference genome [1,7]. In fact, due to cross-fertilization, the genetic diversity of maize is abundant and the linkage disequilibrium (LD) decay is rapid; therefore, this species is a good model for association mapping [8,9].

Association mapping was successfully applied in maize to identify genomic regions related to plant height [4], root development [10], resistance to *Fusarium graminearum* Schwabe [11], and oil biosynthesis in maize kernels [9]. Mapping the associations of chromosomal regions of popcorn kernels revealed the candidate genes for starch, storage protein, pericarp polysaccharide, and oil grain contents in this maize variety [3]. However, little is known about the genes involved in the control of the main popcorn agronomic traits such as grain yield and popping expansion.

Since its inclusion in the germplasm bank of the State University of Northern Rio de Janeiro (UENF), the UENF-14 popcorn population has been subject to a series of recurrent intrapopulational selection cycles targeting the improvement of economically important traits and the maintenance of the population variability. Until the eighth cycle, only phenotypic evaluations were performed [12–20]. The population is currently in the ninth selection cycle (C-9), and the introduction of genotyping proved important for predicting gains for the main traits of interest as well as for allowing a more detailed study of the genomic regions responsible for such traits.

Thus, in the present study, a mixed linear model-based association analysis (MLMA) approach was applied to six agronomic traits of popcorn under different environments to identify potential genes in LD with associated SNPs that might be responsible for these agronomic traits.

## Materials and methods

### Study population

The selected study population belongs to the open-pollinated UENF-14 variety [21]. The original UNB-1 population originated the UNB-2 base population [21], from which the UNB-2U population derived after two mass selection cycles [21]. After five cycles of intrapopulational recurrent selection, the open-pollinated cultivar UENF-14 [21] was released. Currently in its ninth selection cycle, UENF-14 is ideal for association analysis as it is an unstructured population due to the recombinations performed in each recurrent selection cycle [22].

### Phenotypic evaluation of the S_1_ progenies of UENF-14

The S_1_ progenies derived from the UENF-14 population (see below) were subject to two experiments in August 2016: one in Campos dos Goytacazes (ENV1) (northern region of the State of Rio de Janeiro—21°43'15.5"S 41°20'38.3"W), and the other in Itaocara (ENV2) (northwestern region of the State of Rio de Janeiro—21°38'45.2"S 42°03'16.0"W). Each experiment comprised 98 S_1_ progenies, whose parents produced enough seeds to establish these experiments. Both experiments were arranged in incomplete blocks with three orthogonal replications, in which the progenies and eight controls were set. At both environments, the main traits of agronomic interest for this crop were measured: plant height (PH, cm), ear height (EH, cm), 100-grain weight (100GW, g), grain yield (GY, kg/ha), popping expansion of the grain (PE, mL/g), and expanded popcorn volume per hectare (PV, m^3^/ha).

### Genotyping and quality control

Genetic polymorphisms were characterized throughout the genome of 200 plants the open-pollinated UENF-14 in the C-9 cycle. Genomic DNA was extracted from the young leaves of these plants, using the standard CTAB method with modifications (Doyle & Doyle, 1990) and the Capture Seq method [23] was applied with 5000 probes distributed throughout the genome to detect SNP markers. The genotyping procedure generated 21,442 SNPs, to which filters were applied in the following order: a) removal of plants with >10% data loss; b) removal of SNPs with > 5% data loss; and c) removal of SNPs with <0.05 minor allele frequency (MAF). In the study population, 196 plants genotyped with 10,507 SNPs were maintained. The S_1_ progenies referred in the previous sub-section were derived from 98 of these plants.

### Genetic analysis of the population

Based on the 10,507 SNPs and 196 plants, a Genetic Relationship Matrix (GRM) was calculated using the rrBLUP package [24] and the VanRaden algorithm [25]. The same genotypic data set was used to estimate LD, which was measured by the r^2^ statistic calculated for all marker pairs of the same chromosome using PLINK [26]. The LD decay across the genome was analyzed by the nonlinear model proposed by Hill and Weir [27], adjusted with the nlm function of R 3.2.3 [28].

Based on the SNP data for the 196 plants, the absence of population structure was confirmed in STRUCTURE [29]. The algorithm used in this software assumes that the polymorphic locations are in Hardy-Weinberg and linkage equilibriums [29]. Therefore, two extra filters were applied. First, the exact test of Hardy-Weinberg equilibrium removed SNPs with p <0.05. Thereafter, LD pruning was performed for all marker pairs of the same chromosome with r^2^ >0.1, removing one marker and maintaining the other. Both filters were applied using PLINK, resulting in 739 SNPs. The application of the Bayesian model of Pritchard et al. [29] was evaluated according to the criterion of Evanno et al. [30] using the online platform STRUCTURE HARVESTER [31].

### Mixed model-based association analysis

The MLMA [32] was performed for all traits measured at both environments as follows:

$$y=X\beta_{1}+SNP_{i}\beta_{i}+Z_{1}b+Z_{2}u+\varepsilon ,$$ (1)

where **y** is the vector of the phenotypes of a given trait; ***β***_**1**_ the vector of fixed effects including intercept, replication, and co-variables such as the number of plants per plot, counted immediately after thinning, and grain moisture for the traits 100GW, GY, PE, and PV; ***β***_***i*i**_ is the effect of the i^th^ SNP candidate (regression coefficient); ***b*** is the vector of block effects within replications; ***u*** is the vector of polygenic effects; **e** is the vector of residual effects of the model; **X** is the incidence matrix of systematic fixed effects; **SNPi** is the vector of the number of copies of a given allele of the i^-th^ SNP candidate randomly taken as a reference, **SNPi = {0, 1 or 2}**; and **Z**_**1-2**_ are incidence matrices of random effects.

It was also assumed that:

$$b∼N(0,I_{b}\sigma_{b}^{2});u∼N(0,G\sigma_{u}^{2});e∼N(0,I_{n}\sigma_{e}^{2})$$ (2)

and

$$cov(u,b^{\prime })=cov(u,e^{\prime })=cov(b,e^{\prime })=0,$$ (3)

where **G** is GRM calculated using package rrBLUP [24], based on the algorithm of VanRaden [25]; **I**_**b**_ and **I**_**n**_ are the identity matrices in the order equal to the number of incomplete blocks and the number of observations, respectively; $\sigma_{b}^{2},\sigma_{u}^{2},\;and\;\sigma_{e}^{2}$ are the components of variance associated with ***b***, **u**, and **e**, respectively. These components were estimated by the Restricted Maximum Likelihood (REML) method, using the Average Information (AI) algorithm. The MLMA was adjusted using the ASReml-R package [33] as available in R 3.2.3 [28].

To compute the corrected means for the systematic effects, the following model was used:

$$y=X\beta_{1}+Z_{1}b+Z_{2}p+\varepsilon ,$$ (4)

where ***p*** is the progeny effect, taken as fixed for the estimation of the adjusted means (LSMeans); the other terms were as in the previous model. This model was adjusted with package lme4 [34] and the adjusted means were obtained with package lsmeans [35], both as available in R 3.2.3 [28].

The adjusted means were used to estimate the phenotypic correlations between the traits within each environment separately, and for each trait with itself between the two environments. Based on these corrected means, the proportion of the phenotypic variance explained by the markers was estimated using the following model:

$$y_{adj}=\mu +g+e,$$ (5)

where ***y***_***adj***_ corresponds to the adjusted means for a given trait; ***μ*** is the intercept of the model; ***g*** is the genetic component explained by the SNPs; and ***e*** is the random error. This model assumed: $g∼N(0,G\sigma_{g}^{2})$, $e∼N(0,I\sigma_{e}^{2})$, *cov*(*g*,*e*′) = 0, where the ratio of the variance explained by the markers ($h_{m}^{2}$) was calculated as $h_{m}^{2}=\sigma_{g}^{2}/(\sigma_{g}^{2}+\sigma_{e}^{2})$. The model fit for $h_{m}^{2}$ estimation was obtained in REPORT GCTA [36], using the REML method with algorithm AI to estimate the variance components.

### Gene annotation

Genes located in or adjacent to the SNPs within a 100 Kb sliding window (50 Kb to the right and left of the SNP position) were defined as candidate genes for the agronomic traits evaluated. The public dataset of the maize genome and the B73 version 3 reference genome [7] were used. The MaizeGDB genome browser was used for the functional annotations of the candidate genes [37].

## Results

### Characterization of the study population

**Fig 1:**
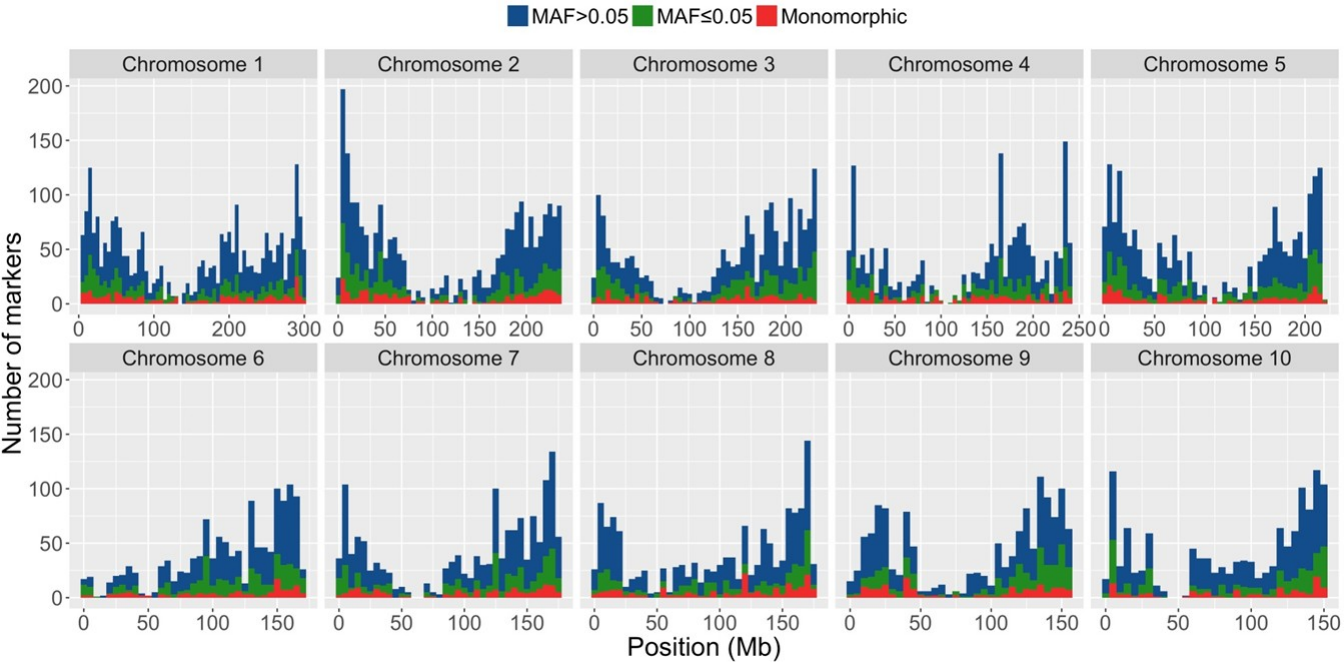
Frequency distribution of monomorphic and polymorphic markers with MAF ≤0.05 and MAF >0.05 throughout the genome. Results correspond to 5-Mb windows.

The frequency distribution of the SNPs throughout the genome was calculated to quantify marker coverage (Fig 1). This evidenced that the polymorphisms across the genome were thoroughly sampled and that the polymorphic markers were well distributed throughout the chromosomes.

To measure the magnitude of the LD and define the windows at which polymorphic locations are expected to be in high LD, the r^2^ among all SNPs belonging to the same chromosome was estimated. The highest LD was observed on chromosome 8, and half of the decay occurred at ~ 151 Kb, while the lowest LD was observed on chromosome 4, where half of the decay was observed at ~ 76 Kb. For all chromosomes, half of the LD was observed at ~ 110 ± 6.82 Kb, on average.

As expected, the absence of population structure was confirmed by the criterion of Evanno et al. [30] (i.e., K = 1 or absence of population stratification) and, when comparing the likelihood function values with the models assuming the different K values on the logarithmic scale (log (L)), the model with K = 1 was deemed the most reasonable.

### Heritability and phenotypic means

The adjusted phenotypic means, as well as the heritability estimates for the SNPs of the six traits evaluated in ENV1 and ENV2, are listed in Table 1. The means of the most important agronomic traits of popcorn were higher in ENV1 (GY = 2,520.42 kg/ha and PE = 28.67 mL/g) than in ENV2 (GY = 2099.94 kg/ha and PE = 27.75 mL/g). In ENV1, the mean values of EH (84.15 cm) and PH (166.97 cm) were lower than in ENV2 (EH = 113.80 cm and PH = 204.13 cm). The values of 100GW were similar at both environments (13.11 g and 13.41 g, respectively), whereas PV was higher in ENV1 (72.55 m^3^/ha) than in ENV2.

**Table 1:** Adjusted phenotypic means and proportions of the mean phenotypic variance explained by the SNP markers ($h_{m}^{2}$), for the six popcorn traits evaluated in ENV1 and ENV2.

Traits	ENV1		ENV2	
	Means	$h_{m}^{2}$	Means	$h_{m}^{2}$
**100GW (g)**	13.11(0.06)	0.29(0.30)	13.41(0.05)	0.36(0.33)
**EH (cm)**	84.15 (0.58)	0.45(0.32)	113.80(0.95)	0.11(0.28)
**GY (Kg/ha)**	2520.42 (42.60)	<0.01(0.25)	2099.94(49.12)	0.37(0.34)
**PE (ml/g)**	28.67 (0.22)	0.43(0.31)	27.75(0.20)	0.74(0.34)
**PH (cm)**	166.97 (0.89)	0.53(0.34)	204.13(1.24)	0.37(0.31)
**PV (m**^**3**^**/ha)**	72.55(1.41)	0.24(0.31)	58.65(1.39)	0.35(0.32)

Values in parentheses indicate the standard error of the estimates. 100GW = 100-grain weight; EH = ear height; GY = grain yield; PE = grain popping expansion; PH = plant height; PV = expanded popcorn volume per hectare.

The proportion of markers explaining phenotypic traits (heritability) was not identical between environments. In ENV1, PH was the trait with the highest value (≅ 53%), while in ENV2, the proportion was highest for PE, with approximately 74% of the SNPs explaining the trait, suggesting that both traits would respond well to SNP marker-assisted selection.

The correlations between the traits in ENV1 were generally very low, ranging from -0.08 (PH vs. PE) to 0.9 (PV vs. GY). In this environment, PV and GY presented the highest correlation (0.9), but a high value was also found for the correlation between PH and EH (- 0.82; Table 2). Both of these correlations were significant (p <0.01).

**Table 2:** Phenotypic correlations between the traits evaluated within and between environments (ENV1 and ENV2).

	ENV1					
ENV2	100GW	EH	GY	PE	PH	PV
**100GW**	**0.71(<0.01)**	0.21(0.04)	0.18(0.08)	-0.07(0.47)	0.29(<0.01)	0.13(0.21)
**EH**	0.19(0.06)	**0.83(<0.01)**	0.20(0.05)	-0.08(0.41)	0.82(<0.01)	0.13(0.2)
**GY**	0.24(0.02)	0.23(0.02)	**0.34(<0.01)**	0.12(0.25)	0.18(0.08)	0.9(<0.01)
**PE**	-0.08(0.43)	0(0.98)	0.12(0.24)	**0.48(<0.01)**	-0.08(0.45)	0.52(<0.01)
**PH**	0.23(0.02)	0.83(<0.01)	0.22(0.03)	0(0.97)	**0.8(<0.01)**	0.12(0.25)
**PV**	0.19(0.06)	0.23(0.03)	0.93(<0.01)	0.45(<0.01)	0.22(0.03)	**0.31(<0.01)**

Upper diagonal: trait correlations in ENV1; lower diagonal: trait correlations in ENV2. Central diagonal: trait correlations at both environments. 100GW = 100-grain weight; EH = ear height; GY = grain yield; PE = popping expansion; PH = plant height; PV = expanded popcorn volume per hectare.

In ENV2, trait correlations ranged from -0.08 (PE vs. 100GW) to 0.93 (GY vs. PV). Although the highest values were observed GY and PV, the correlation between EH and PH was also high and significant (- 0.83, p <0.01; Table 2). In both environments, the correlation between GY and PE was 0.12.

The correlations between the traits in the two environments ranged from 0.31 (PV) to 0.83 (EH), and all associations were significant (P <0.01).

### Mixed model-based association analysis

**Fig 2:**
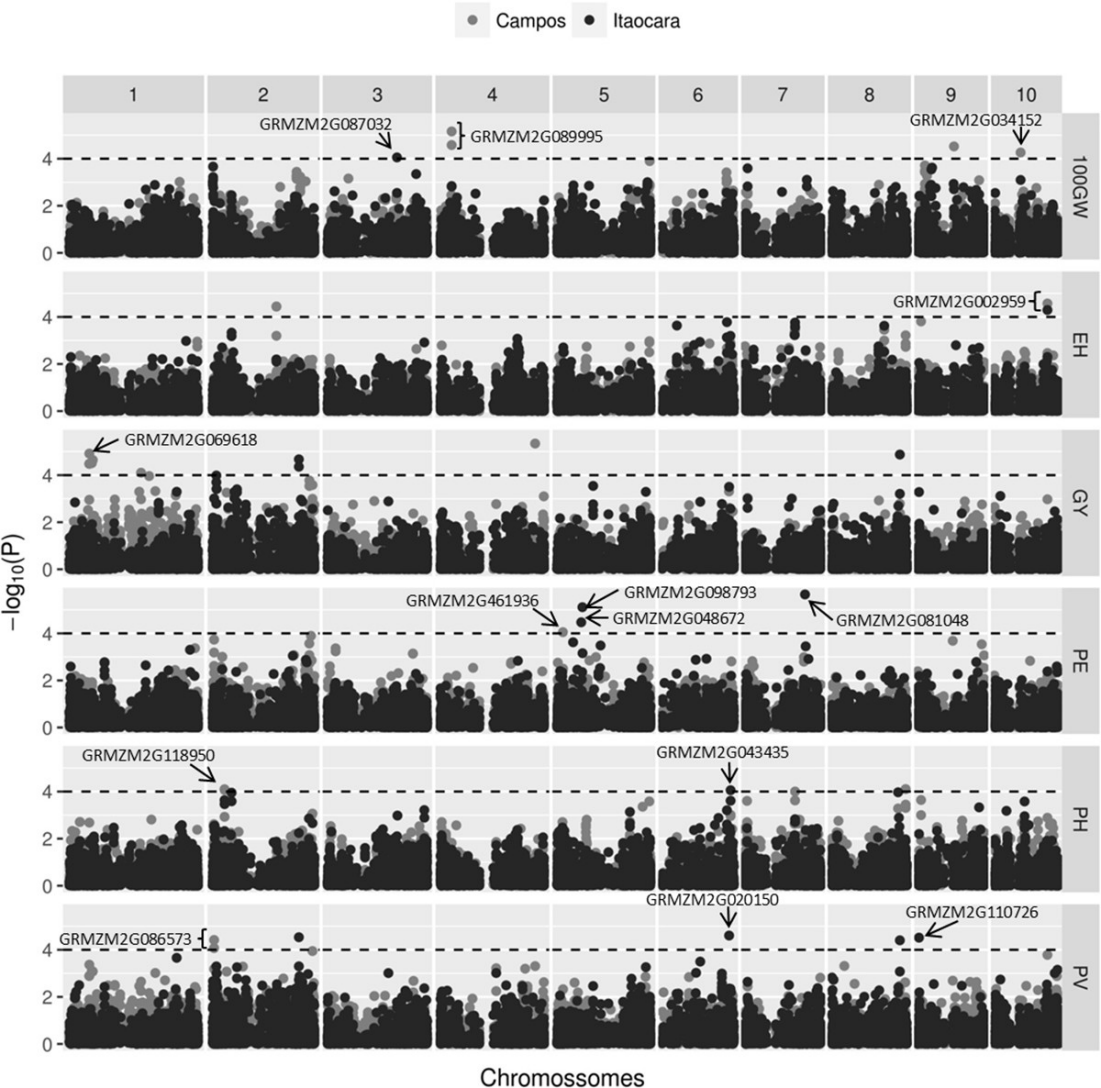
Manhattan plot resulting from the mixed linear model-based association analysis of SNP markers (n = 10,507) in the environments of Campos dos Goytacazes (ENV1) and Itaocara (ENV2). Significant SNP markers are shown above the dotted lines (p ≤0.0001). Arrows indicate the candidate genes according to the MaizeGDB genome.

To find the genomic regions associated with traits of interest in popcorn, MLMA was performed. The Manhattan plot obtained (Fig 2) revealed the significance of association for all SNPs (n = 10,507; p ≤0.0001, or -log10 (p) ≥4). Based on the LD results, a search window was established for the annotated genes, possibly in high LD, and their associated SNPs. Thus, a 100 Kb window was standardized, corresponding to 50 Kb to the right and left of each SNP. The candidate genes flanking the genetic associations that were determined by MLMA in ENV1 and ENV2 are shown in S1 Table and S2 Table, respectively.

By comparing the physical locations of significant SNPs in the B73 reference genome, the candidate genes were identified. In ENV1, the genes were distributed on chromosomes 1, 2, 4, 5, and 10. For EH, the gene GRMZM2G002959 (S1 Table) coding for glutaryl-CoA dehydrogenase was identified, although its function in plant species could not be identified. The gene GRMZM2G089995 (S1 Table) was identified for 100GW, and it encoded the APETALA2/ETHYLENE-RESPONSIVE ELEMENT BINDING PROTEINS (AP2/EREBP) transcription factor 209, related to plant responses to abiotic stresses [38]. The gene GRMZM2G034152, encoding the protein polyamine oxidase (PAO1), was also identified for 100GW, and it was related to biotic or abiotic stress response [39]. Gene GRMZM2G069618), encoding a tetrapeptide-containing protein (TRP), was identified for GY (S1 Table), and it is involved in plant stress, hormone signaling [40], and root development [41]. Gene GRMZM2G461936, encoding ARGONAUTE108 (AGO108), was identified for PE. Argonaute proteins (AGO) function in cooperation with micro RNAs (miRNAs) or small interfering RNAs (siRNAs) and regulate gene silencing at the post-transcriptional level [42]. Gene GRMZM2G118950, which encodes AMMONIUM TRANSPORTER3 (AMT3) related to ammonium uptake from the soil solution [43], was identified for PH. Gene GRMZM2G086573 (S1 Table), encoding the AP2/EREBP transcription factor 24 was identified for PV. The AP2/EREBP transcription factors are found extensively in plants and are involved in their growth, development, and signal transduction of numerous physiological and biochemical responses, including floral organogenesis, seed development, carbon metabolism, and pathogen resistance, among others [44].

In ENV2, the genes were distributed on chromosomes 3, 5, 6, 7, 9, and 10 (S2 Table). Gene GRMZM2G002959 (S2 Table), encoding glutaryl-CoA dehydrogenase with an unknown function in plants, was identified for EH. Gene GRMZM2G087032 (S2 Table), encoding the putative bifunctional protein C3H transcription factor 313, which may be important during the initial stage of maize seed filling [45], was identified for 100GW. Gene GRMZM2G098793 (S2 Table), encoding the superfamily of glycosyltransferase enzymes that are responsible for glycosylation, a fundamental mechanism for determining the chemical complexity and diversity of natural plant products [46], was identified for PE. Genes GRMZM2G081048 and GRMZM2G048672 (S2 Table), the first encoding an oxidoreductase and the second a macrophage migration inhibitory factor, were also associated with PE. Oxidoreductases catalyze the electron transfer from one (reducing) molecule to another (oxidant) and play important roles, not only in electron transfer, but also in several biosynthetic processes and biodegradation pathways [47]. The macrophage migration inhibitory factor is a cytokine found in humans [48], whose function in plants is not yet identified. Genes GRMZM2G110726 and GRMZM2G020150 (S2 Table) were associated with PV. The first is related to protein BOBBER1, also found in *Arabidopsis thaliana* L., and responsible for limiting the extension of the meristem and/or the development of the cotyledon domains [49], as well as for developmental and thermo-tolerance functions [50]. The second, encodes the AP2/EREBP transcription factor 196, which is involved in growth and development, as well as in other important functions in plant organisms [44]. No gene was identified as related to GY.

## Discussion

### Population structure

The LD decay varied between the chromosomes (76–151 Kb), which is in agreement with the mean LD decay range observed for 64 maize lines (80–100 kb) [51]. As expected, the criterion of Evanno et al.[30] was sensitive enough to test K = 1 (absence of subpopulations). These results indicate that the studied population is not highly stratified and its possible light structure may be explained by the random common alleles received during the several recombination cycles.

### Association analysis

Several genes might be present in the range of an associated region (i.e., near SNPs), and these can be identified by MLMA. This analysis identified seven and eight genes related to the studied traits in ENV1 and ENV2, respectively. Gene GRMZM2G002959, identified for EH in both environments encodes glutaryl-CoA dehydrogenase but its function in plant species has not been identified.

Gene GRMZM2G089995, identified for the 100GW in ENV1, encodes the transcription factor 209, a member of the AP2/EREBP protein family, which is directly involved in the response to biotic and abiotic stresses [52–54]. For the same trait, gene GRMZM2G034152 encoding PAO1, whose function is also related to responses to abiotic stresses in rice [39] and citrus [55], and to biotic stresses in cotton [56], was identified. This indicated that 100GW is related to stress responses (biotic and abiotic). This agrees with previous results obtained for drought-stressed rice, in which a reduction in 1000-grain weight [57] was observed, and to the premise that drought stress contributed to a yield reduction in rice lines [58]. In soybean, seeds grown under adverse climatic conditions also had a lower 100-grain weight [59].

In ENV2, the candidate gene GRMZM2G087032, identified for 100GW, corresponds to the putative bifunctional protein C3H transcription factor 313, which may be important during the initial stage of maize grain filling [45]. However, the study of this protein is still at an early stage, as the research on this topic is very limited.

In ENV1, the candidate gene GRMZM2G069618, related to GY, was found on chromosome 1. It encodes a protein containing tetratricopeptide repeats (TPR) that is involved in plant stress and hormone signaling [40], and that is responsible for root development, as deficiency of the SSR1 gene (encoding TRP) negatively affects the transport of auxin [41], an essential plant hormone regulating root growth [60]. Poor root formation can damage the developing grain, by preventing the uptake of nutrients for grain formation.

In ENV1, the candidate gene GRMZM2G461936, which encodes AGO108, was identified as being related to PE. Argonaute proteins are fundamental in the regulation of gene expression and are essential for several developmental processes [61]. These authors identified the protein ZmAGO18b in maize genotypes and observed a high expression of this protein in reproductive tissues. Although no studies have specifically identified AGO108 in plants, deficiency in AGO10 induced an abnormal development of the apical meristem of buds in *A*. *thaliana* plants [62]. Gene GRMZM2G098793, encoding a member of the glycosyltransferase enzyme superfamily, was also identified as related to PE in ENV2. In wheat endosperm starch, the amount of arabinoxylan, which is one of the most abundant polysaccharides, was reduced after the suppression of two glycosyltransferase homologous genes [63]. This suggested that the gene identified in the present study is also involved in starch synthesis in popcorn kernels, and that its suppression can modify the endosperm, whose function is directly related to the grain expansion capacity under high temperatures. Gene GRMZM2G081048, also associated with PE, encodes the enzyme oxidoreductase. In the present study, it was not possible to identify which type of oxidoreductase is associated with the trait, impairing a more in-depth analysis of its functions. Gene GRMZM2G048672, also identified as related to PE, was shown to encode the immunoregulatory cytokine macrophage migration inhibitory factor. However, no reports of its function in plants were found in the literature.

In ENV1, the gene GRMZM2G118950 identified for PH encodes AMT3. Ammonium transporters are responsible for ammonium uptake from the soil solution [43,64]. Two AMTs located in the rhizodermis (ZmAMT1;1a and ZmAMT1;3) identified in a previous study are probably the principal components of the high affinity ammonium transport system in maize roots [65]. In maize, ammonium has several beneficial effects, such as the increase of root density and extension [66]. Therefore, ammonium uptake may directly influence PH.

In ENV2, gene GRMZM2G043435, encoding the respiratory burst oxidase-like protein C (RbohC), was associated with PH. In *A*. *thaliana*, RbohC-deficient mutants had short root hair on stunted roots, suggesting that RbohC regulates plant cell expansion [67] and might be involved in height development of popcorn plants.

One gene (GRMZM2G086573) was identified as related to PV in ENV1. This gene encodes the AP2/EREBP transcription factor 24. The distinctive feature of AP2/EREBP proteins is that they contains one or two AP2 domains, which are binding elements to the response element ethylene. The rice starch regulator gene (*RSR1*), a transcription factor of the APT2 family, negatively regulates the expression of type I starch synthesis genes as *RSR1* deficiency resulted in the increased expression of starch synthesis genes in rice seeds [68]. Overall, PV depends on the grain moisture and starch content, which is converted into steam and exerts pressure on the endosperm [69].

In ENV2, two genes were identified as associated with PV: GRMZM2G110726, which encodes the protein BOBBER 1, also found in *A*. *thaliana*, and responsible for limiting the extension of the meristematic domain and/or promoting the development of the cotyledon domains [49], as well as participating in developmental and thermo-tolerance functions [50]; and GRMZM2G020150, encoding the protein AP2/EREBP transcription factor 196. Similar to the other transcription factor of the APT2 family found in rice, this might be responsible for negatively regulating the expression of genes of type I starch synthesis [68], which are correlated with the capacity of the grain to expand, as this depends directly on the starch content of the grain [69].

### Genomic relationships between candidate genes

There were no significant common markers between the analyzed agronomic traits. Despite the high correlation between GY and PV (0.93, p<0.01), no SNP was common to both traits. However, these traits are directly related because PV is calculated based on GY and PE. Thus, PV should be used as a "super trait" in recurrent popcorn selection programs, as its inclusion results in optimized simultaneous gains for GY and PE [19,70]. Although PH and EH were slightly correlated in both environments (0.82 and 0.83; Table 2), the significant genomic regions for the two traits did not overlap. The QTLs controlling the two traits were identified and there was no QTL controlling any single trait independently [71].

The increased number of functional assays and the higher number of evaluated traits will produce more available data for the different populations, which will be extremely useful to address important biological issues. The MLMA strategy used in the present study proved useful and robust, complementary to biparental cross-mapping, and can map multiple genetic traits simultaneously [72]. The main objective of association analyses is to identify genes related to traits of interest. Thus, the genomic regions and the traits of interest must be strongly associated to allow the identification of trait-related SNPs. The SNPs identified here are extremely important for accelerating the breeding process using marker-assisted selection, and can be incorporated in genomic selection strategies.

## Conclusion

The initial stimulus for this study was the fact that, to date, few studies have addressed the genetic architecture and mechanisms that control the natural variation in the development and productivity of popcorn genotypes. The results obtained here showed that some morphological traits are moderately inheritable, such as PH and EH, with wide variation in a population containing different lines genotyped with 10,507 SNPs. The candidate genes associated to these loci are an inestimable resource for gene function analyses and for dissecting the molecular network regulating the development of popcorn traits, apart from identifying genome polymorphisms, which are essential in marker-assisted selection of agronomic traits in breeding programs. Twelve proteins were identified as being SNP-associated, providing new information useful for accelerating popcorn breeding programs, as this crop accounts for an annual turnover of about one billion dollars in the United States.

## Supporting information

S1 Table Candidate genes obtained from the SNP-based association analysis for six traits of interest in popcorn–ENV1. (DOCX)

S2 Table Candidate genes obtained from the SNP-based association analysis for six traits of interest in popcorn–ENV2. (DOCX)

S3 Table Detailed information of SNPs used in the study (SNP name, chromosome, physical position (bp) and polymorphic alleles). (XLS)

S4 Table Summary of statistical analysis for key traits of popcorn measured in a two environments (Campos dos Goytacazes and Itaocara). (XLS)
