# Supplementary material for: SNP-based mixed model association of growth- and yield-related traits in popcorn
Source: PLoS One. 2019 Jun 25;14(6):e0218552. doi: 10.1371/journal.pone.0218552 (PMC6592533; doi:10.1371/journal.pone.0218552)
Supplement: S1 Table — (DOCX) [file pone.0218552.s001.docx]

**S1 Table**. Candidate genes obtained from the SNP-based association analysis for six traits of interest in popcorn - ENV1.

| **Nº** | **Trait** | **SNP ID** | **Chr*** | **Position (bp)** | **MAF ^a^** | **P value** | **Annotation** |
| --- | --- | --- | --- | --- | --- | --- | --- |
| 1 | EH | GRMZM2G002959 | 10 | 124300563 | 0.255102 | 0.0000269 | Glutaryl-CoA dehydrogenase |
| 2 | 100GW | GRMZM2G089995 | 4 | 26424338 | 0.1173469 | 0.0000069 | AP2/EREBP-transcription factor 209 |
| 3 | 100GW | GRMZM2G034152 | 10 | 62249986 | 0.2857143 | 0.0000546 | Polyamine oxidase 1 |
| 4 | GY | GRMZM2G069618 | 1 | 56893602 | 0.0688776 | 0.0000228 | TPR domain containing protein |
| 5 | PE | GRMZM2G461936 | 5 | 13615937 | 0.2959184 | 0.0000889 | AGO-108 - argonaute108 |
| 6 | PH | GRMZM2G118950 | 2 | 28937135 | 0.1173469 | 0.0000793 | AMT-3 - ammonium transporter3 |
| 7 | PV | GRMZM2G086573 | 2 | 4674885 | 0.1572165 | 0.0000383 | AP2/EREBP-transcription factor 24 |

* Chromosome

^a^ Minor allele frequency
