# Supplementary material for: SNP-based mixed model association of growth- and yield-related traits in popcorn
Source: PLoS One. 2019 Jun 25;14(6):e0218552. doi: 10.1371/journal.pone.0218552 (PMC6592533; doi:10.1371/journal.pone.0218552)
Supplement: S2 Table — (DOCX) [file pone.0218552.s002.docx]

**S2 Table.** Candidate genes obtained from the SNP-based association analysis for six traits of interest in popcorn - ENV2

| **Nº** | **Trait** | **SNP ID** | **Chr*** | **Position (bp)** | **MAF ^a^** | **P value** | **Annotation** |
| --- | --- | --- | --- | --- | --- | --- | --- |
| 1 | EH | GRMZM2G002959 | 10 | 124300563 | 0.255102 | 0.0000509 | Glutaryl-CoA dehydrogenase |
| 2 | 100GW | GRMZM2G087032 | 3 | 161700883 | 0.0535714 | 0.0000880 | C3H-transcription factor 313 |
| 3 | PE | GRMZM2G098793 | 5 | 58753471 | 0.1147959 | 0.0000077 | Glycosyltransferase |
| 4 | PE | GRMZM2G081048 | 7 | 139116617 | 0.1096939 | 0.0000022 | Oxidoreductase |
| 5 | PE | GRMZM2G048672 | 5 | 56022372 | 0.0994898 | 0.0000339 | Macrophage migration inhibitory factor |
| 6 | PH | GRMZM2G043435 | 6 | 160035981 | 0.0807292 | 0.0000875 | Respiratory burst oxidase-like protein C |
| 7 | PV | GRMZM2G110726 | 9 | 3937091 | 0.2602041 | 0.0000305 | Protein BOBBER 1 |
| 8 | PV | GRMZM2G020150 | 6 | 156592891 | 0.4540816 | 0.0000248 | AP2/EREBP-transcription factor 196 |

* Chromosome

^a^ Minor allele frequency
